# Community case study: an academia-industry-government partnership that monitors and predicts outbreaks in Tri-County Detroit area since 2017

**DOI:** 10.3389/fpubh.2024.1475425

**Published:** 2025-01-28

**Authors:** Irene Xagoraraki, Liang Zhao, Yabing Li, Brijen Miyani, John Norton, James Broz, Andrew Kaye, Anna Mehrotra, Anil Gosine, Scott Withington, Stacey McFarlane, Russell A. Faust

**Affiliations:** ^1^Department of Civil and Environmental Engineering, Michigan State University, East Lansing, MI, United States; ^2^Great Lakes Water Authority, Detroit, MI, United States; ^3^CDM Smith, Detroit, MI, United States; ^4^Water Environment Federation, Washington, DC, United States; ^5^Detroit Water and Sewerage Department, Detroit, MI, United States; ^6^Detroit Health Department, Detroit, MI, United States; ^7^Macomb County Health Division, Mount Clemens, MI, United States; ^8^Oakland County Health Division, Pontiac, MI, United States

**Keywords:** wastewater surveillance, prediction, screening, SARS-CoV-2, communicable diseases, academia-industry-government partnership

## Abstract

The Tri-County Detroit Area (TCDA) is the 12th most populous metropolitan area in the United States with over three million people. Multiple communicable diseases are endemic in the TCDA. In 2017, to explore innovative methods that may provide early warnings of outbreaks affecting populations in the TCDA, an exploratory partnership that was funded by a U.S. National Science Foundation Early-concept Grant for Exploratory Research (EAGER) began. Since 2017, a project team including the College of Engineering at Michigan State University (MSU), the City of Detroit, the Great Lakes Water Authority (GLWA), industry, and local government and health departments, has been testing municipal wastewater from the TCDA to survey and predict surges in communicable diseases in the area. This ongoing effort started years before wastewater-based epidemiology became a widespread method in public health practice, due to the COVID-19 pandemic, and is now supported by the U.S. Centers for Disease Control and Prevention (CDC). The work of the partnership led to significant breakthroughs in the field of wastewater surveillance/wastewater epidemiology. The results of our surveillance efforts are used to assist local health departments in their understanding and response efforts for health issues in the TCDA, facilitating public health messaging for local awareness, targeted clinical testing, and increased vaccination efforts. Our data are available to the local health departments, and our methodological advancements are published and have been used by other communities nationwide and beyond. This paper describes the partnership, lessons learned, significant achievements, and provides a look into the future. The successful implementations and advancements of wastewater surveillance in the TCDA advocate the importance of frequent communications and interactions within the partnership, idea generations from each stakeholder for decision-making, maintenance of scientific rigor, ethical awareness, and more.

## 1 Introduction

This project focuses on early detection of emerging outbreaks and understanding of temporal and special fluctuation of communicable disease in the Tri-County Detroit Area (TCDA) population. To rapidly, and accurately, identify novel and emerging infectious diseases and to predict impending outbreaks, sophisticated metrics and their dynamic interrelationships must be effectively modeled. Traditional infectious disease detection systems, which are predicated on the diagnostic analysis of clinical specimens, have significant limitations for predictive intelligence. Patients are generally only examined in a clinical setting after symptom onset, often after the commencement of a disease surge in their community. Screening all, or even a statistically appropriate proportion, of individuals in communities at a regular cadence for every potentially emerging, rare, and novel pathogen would be practically impossible. Municipal wastewater (a mixture of water from the sink, toilet, shower, dishwasher, laundry, and often stormwater and industrial water) surveillance offers a pragmatic and reliable method for obtaining community composite samples (CCSs). Combined with novel genomic testing and multidata process modeling, municipal wastewater surveillance provides accurate early warnings of disease outbreaks prior to detection at clinical settings.

The on-going partnership in the Tri-County Detroit Area (TCDA) started 3 years before the COVID-19 pandemic which brought wastewater-based epidemiology to the forefront of public health surveillance. Since the start of the pandemic, municipal wastewater surveillance has been increasingly used in the U.S., as evidenced by the establishment of the National Wastewater Surveillance System (NWSS) by the U.S. Centers for Disease Control and Prevention (CDC). Through NWSS, CDC supports federal, state, and local government surveillance efforts to monitor community transmission of SARS-CoV-2, influenza A and B, respiratory syncytial virus (RSV), and other pathogens (1). Efforts by the TCDA partnership described here have provided key contributions to wastewater-based epidemiology methodology and practice in the U.S., that are explicitly described in this paper.

We identified that our multi-stakeholders’ partnership was critical in successfully implementing, maintaining, and expanding local wastewater surveillance programs. Briefly, frequent knowledge sharing and routine weekly updates through online meetings and email communications, collective solutions and decision-making through team discussions, active participations of multi-stakeholders, all facilitated the establishment of wastewater surveillance as an effective and functioning public health measure in the TCDA. In this paper, we describe the interactive academia-industry-government partnership and its journey from an unconventional method to a nationally accepted approach.

## 2 Methods

### 2.1 The focus area

Most of the population of the TCDA is served by the Great Lakes Water Authority (GLWA) Water Resource Recovery Facility (WRRF) that is the largest single-site wastewater treatment plant in the U.S. that treats wastewater from an estimated 3.2 million inhabitants. The WRRF serves the three largest counties in Michigan, including Wayne County (1.8 million inhabitants), Oakland County (1.2 million), and Macomb County (0.8–0.9 million) in an area of more than 946 square miles covering 76 communities (2, 3). Wastewater from residents in the service area flows to three distinct interceptors.

Since 2017, we have been collecting and analyzing wastewater from all three interceptors and the results represent disease trends in the whole population of the TCDA. In 2020, in addition to the three centralized interceptor locations, we also began collecting wastewater from street manholes in nine localized neighborhood sewersheds in Wayne, Macomb, and Oakland counties. Locations of the nine sewersheds were selected to encompass communities with different population sizes and diverse demographic characteristics, including varying racial composition, poverty percentages, income and education levels (4).

### 2.2 The partnership

**Figure 1:**
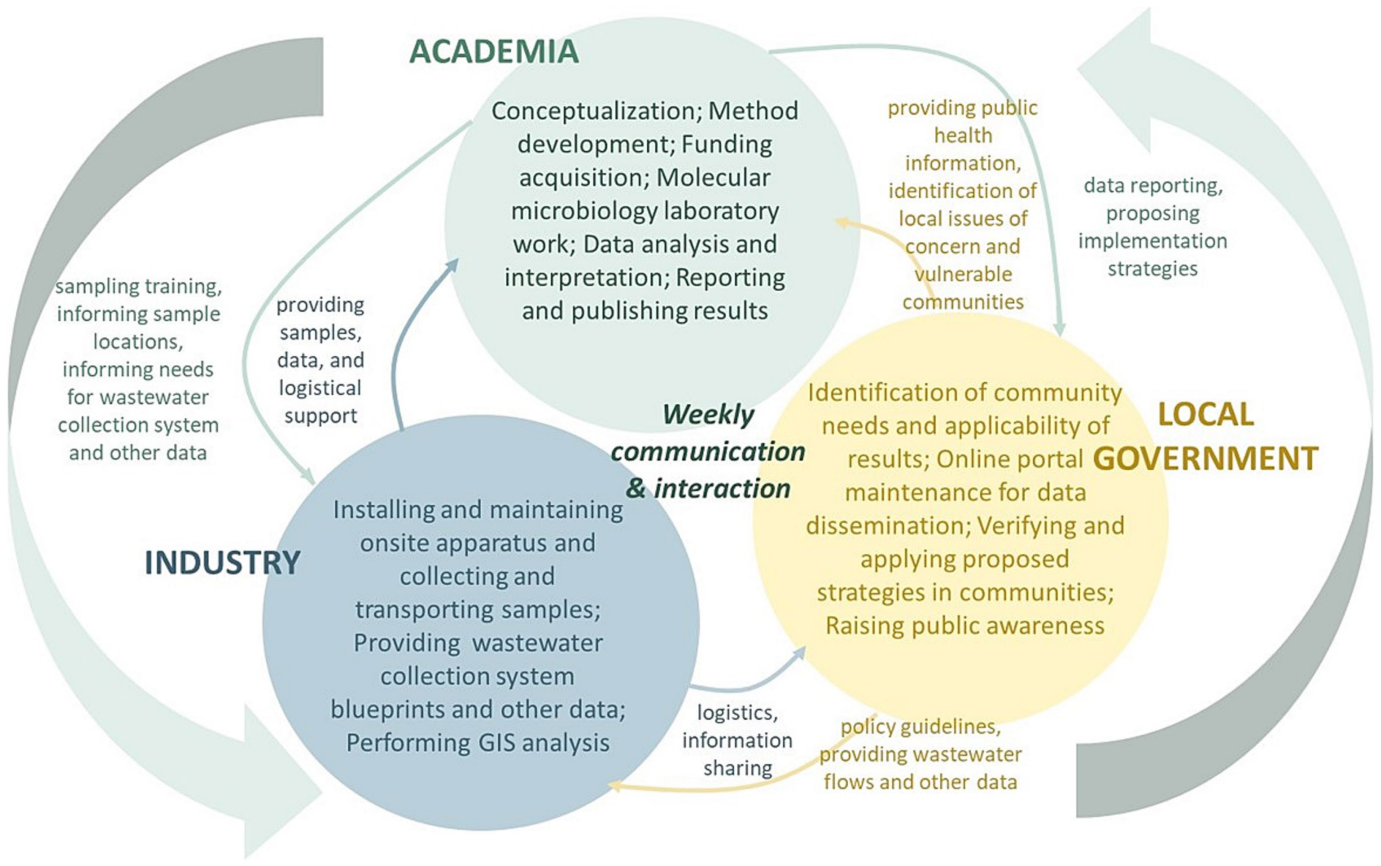
Tri-County Detroit area (TCDA) wastewater surveillance partnership (WSP) interactions.

The partnership includes academia (College of Engineering at Michigan State University), industry (Great Lakes Water Authority and CDM Smith) and government (Detroit Water and Sewerage Department, Detroit Health Department, Oakland County Health Division, Macomb County Health Department, and Michigan Department of Health and Human Services). Briefly, frequent knowledge sharing and routine weekly updates through online meetings and email communications, collective solutions and decision-making through team discussions, active participations of multi-stakeholders, all facilitated the ongoing success of this project and the establishment of wastewater surveillance as an effective and functioning public health measure in the TCDA. Each sector within the academia-industry-government partnership plays an indispensable role (Figure 1).

**Figure 2:**
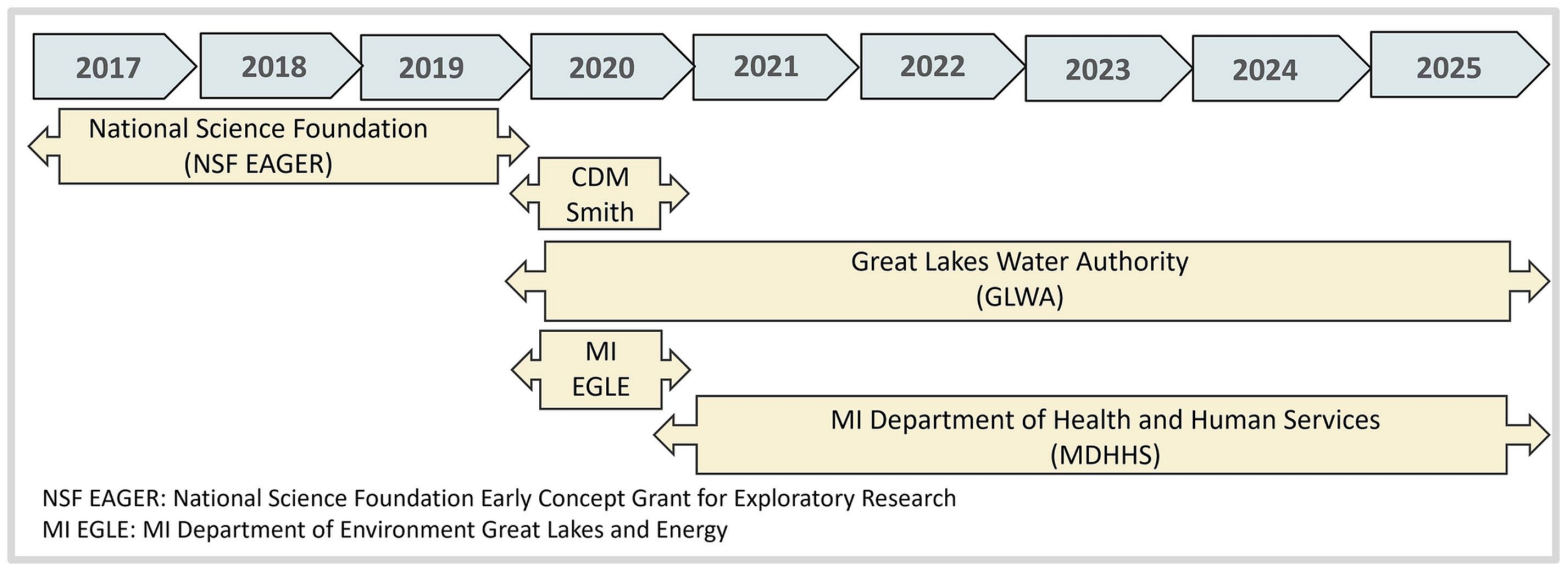
TCDA-WDP funding evolution.

A summary of task distributions is shown in Figure 2. Health departments are best positioned to evaluate the effectiveness and value of information generated by wastewater surveillance, which enables them to implement public health responses and strategies in real world applications. Researchers in academia have expertise in developing and advancing methodologies, processing samples that are collected by the industry partners, analyzing and interpreting data, providing information to health departments. The local municipality and the industry sector of the partnership, including GLWA, and CDM Smith, input critical evidence to better understand practicality of sampling methods, locations, transportations, and other sampling-related logistics issues. Notably, failing to include any sector within the consortium would have severely limited successful development, implementation, and maintenance of a thriving surveillance program.

Decisions are made in the partnership based on input and knowledge sharing from, and active participation of, all stakeholders. In addition to weekly meetings, partnership members share news, updates, and issues via emails, and are regularly collaborators on research manuscripts.

### 2.3 Funding

**Figure 3:**
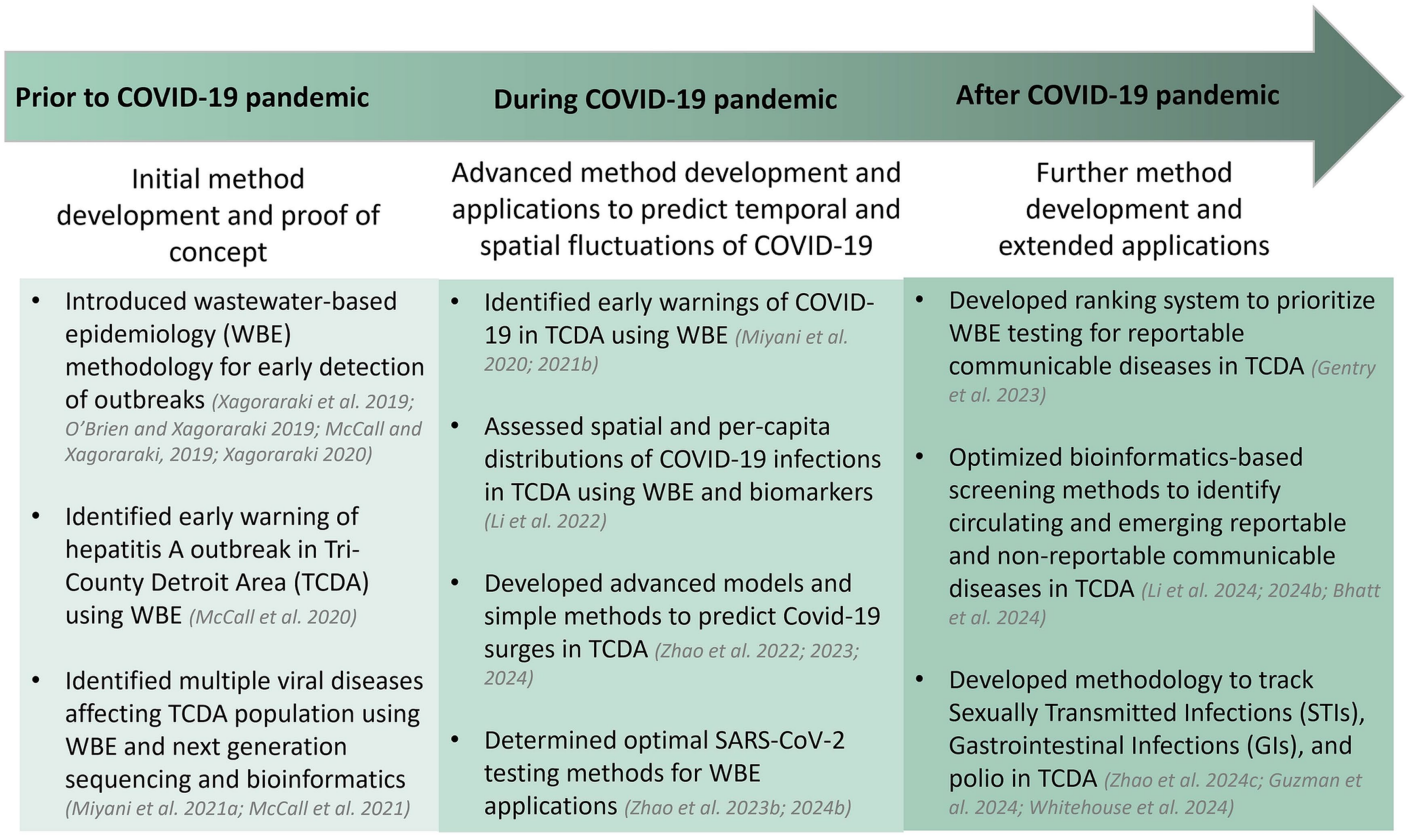
TCDA-WSP phases and outcomes.

The project started with a modest $150,000 National Science Foundation Early Concept Grant for Exploratory Research (NSF EAGER) awarded in 2017. According to NSF, the EAGER funding mechanism supports exploratory work in its early stages on untested, but potentially transformative, research ideas or approaches. It funds work that could be considered especially “high risk, high payoff” in the sense that it involves radically different approaches, applies new expertise, or engages novel disciplinary or interdisciplinary perspectives. To this date the project has received over $7 M from the Michigan Department of Health and Human Services, $0.8 M from the Michigan Department of Environment Great Lakes and Energy and over $ 0.5 M from GLWA (Figure 3).

## 3 Results

**Figure 4:**
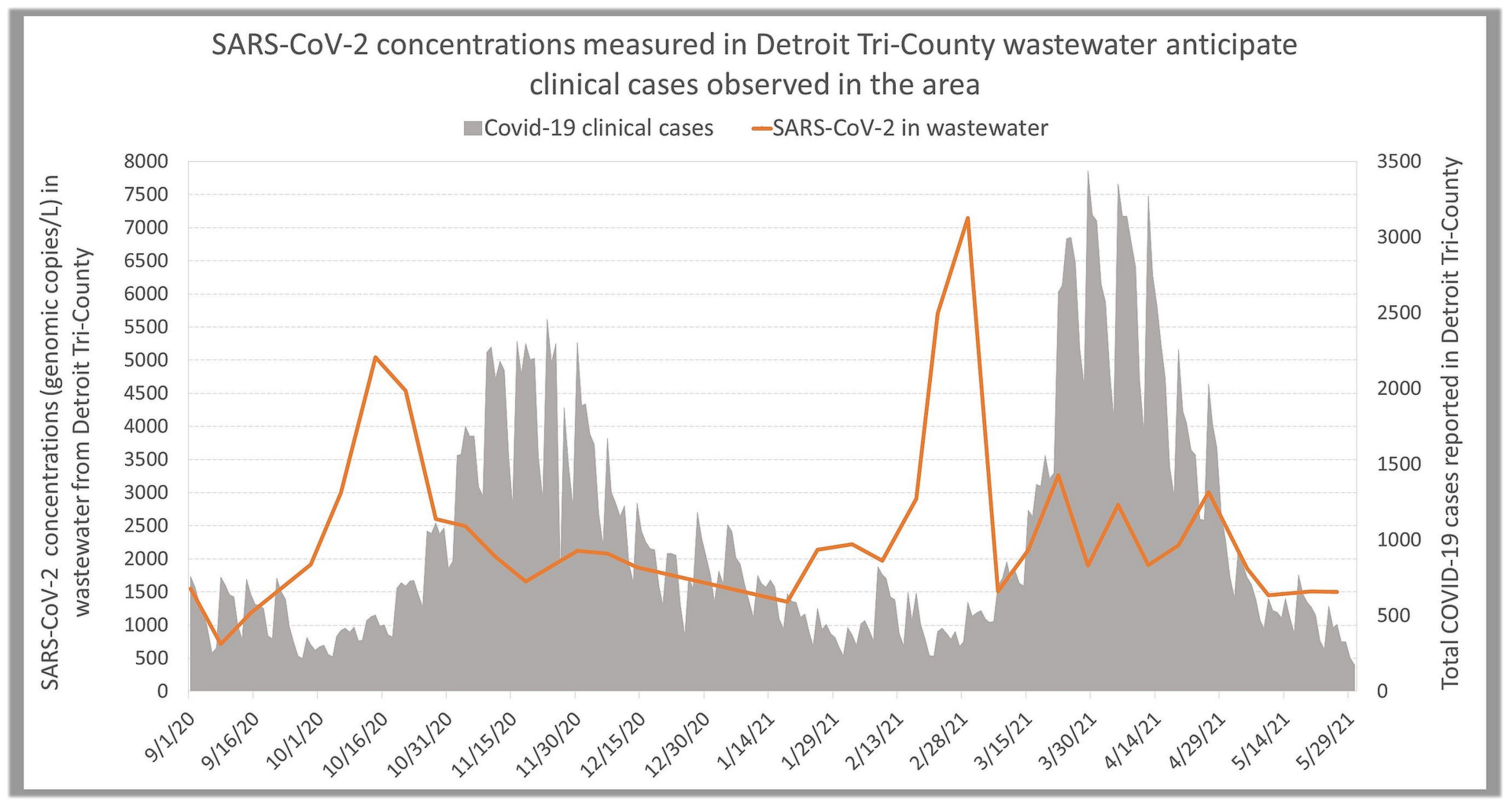
Wastewater surveillance predicts peaks of Covid-19 cases [more data and methods shown at Zhao et al. (17)].

The timeline of the TCDA partnership outcomes prior to, amid, and beyond the COVID-19 pandemic is presented in Figure 4.

### 3.1 Prior to Covid-19 pandemic

The initial effort (5, 6) that began in 2017 resulted in the introduction of wastewater-based epidemiology methodological approaches (including laboratory and data analysis methods) that lead to early warnings of infectious disease outbreaks, prediction of temporal variations of clinical cases, as well as investigations of spatial fluctuations of disease (7–13).

We applied the methodology to identify several enteric viruses circulating in the area, and identified hepatitis A outbreak peaks that occurred in the Detroit area population during 2017–18 (8). We detected multiple human viruses, including enteric, respiratory, bloodborne, and vector-borne viruses, excreted by the population, associated with reportable and non-reportable viral diseases circulating in the Detroit community during 2017–18 (7). In a further study we indicated the presence of nine types of human herpesviruses in wastewater (12), with human herpesvirus 8 being the most abundant possibly related to the HIV-AIDS outbreak that was occurring in the Metro Detroit Area during the study period. We developed and used innovative next-generation sequencing and bioinformatics-based screening methodologies that allow identification of circulating communicable diseases in the community beyond the reportable diseases (7, 8, 12).

### 3.2 During Covid-19 pandemic

In 2020, as an effort to combat COVID-19, the exploratory work shifted to monitor SARS-CoV-2 in wastewater (14) and has been funded by the MDHHS and by GLWA ever since. Our measurements are reported to the local health departments and are published on the Michigan COVID-19 Wastewater Testing Dashboard (15). SARS-CoV-2 measurements in wastewater provided early warnings of COVID-19 clinical cases, especially during the initial stages of the pandemic. As the virus mutated and the incubation time decreased, the early warning potential of wastewater measurements decreased. However, during the latest stages of the pandemic, clinical data reporting reduced significantly, and asymptomatic infections likely increased, therefore wastewater surveillance maintained its value in tracking COVID-19 cases in the population (16).

Early in the pandemic, we were able to identify COVID-19 peaks in clinical cases in the TCDA 5 weeks prior to clinical data peaks when the SARS-CoV-2 Delta variant was occurring (Figure 4) using advanced statistical models (17). Further, we developed simple statistical methodology to determine early warnings of COVID-19 surges that are used for informed decision making by public health officials (16). We also compared time series data of wastewater measurements and clinical data to optimize early warning potential of different wastewater concentration methods (18), and tracked the time lag between SARS-CoV-2 concentrations in wastewater and diverse clinical metrics through time lagged cross correlation methods (19). Subsequently, we developed experimental and statistical methodology to compare three commonly applied CDC assays targeting SARS-CoV-2 in wastewater, including N1, N2 and SC2 assays, thereby identifying N2 assay as the optimal target to predict COVID-19 cases in Detroit’s wastewater (20). To allow for comparisons between communities (4), the team used metabolites and biomarkers (such as total Kjeldahl nitrogen, creatinine, and xanthine) as well as wastewater quality measurements to normalize the SARS-CoV-2 measurements.

### 3.3 After Covid-19 pandemic

To expand wastewater surveillance beyond SARS-CoV-2, we created a ranking method that incorporates clinical data trends and other parameters to prioritize pathogens that should be targeted in specific geographic locations (21). The Communicable Diseases Wastewater Surveillance Ranking system (CDWSRank) is based on 6 binary parameters and 6 quantitative parameters (21). Binary parameters include the presence or absence of reportable communicable diseases in multiple governmental lists and databases, the pathogen’s detectability in wastewater or human excrement, and the association of disease with single or multiple pathogens. Quantitative parameters include regional and state-level clinical case trends, local and county-level clinical case trends, geographic ratio of clinical case incidence locally and regionally, the basic reproduction number (R_0_) of the disease, etc. An overall ranking score of the CDWS-Rank system for reportable communicable diseases is calculated involving these parameters and weighing factors. The ranking system provides a data-driven approach to public health decision-making and promotes broader applications of wastewater surveillance for public health benefits (21). It helps health departments, funding agencies, researchers and practitioners to prioritize resources and efforts toward monitoring emerging community diseases using wastewater surveillance.

Using the CDWSRank system, the reportable diseases that were prioritized for wastewater surveillance in TCDA included diseases that are caused by viruses *(COVID-19, Hepatitis B, Measles, Influenza, Hepatitis C, Varicella zoster, Chickenpox, Dengue, Mumps, Polio, HIV-AIDS, Hepatitis E, Shingles, Rubella, Norovirus)* and bacteria *(Gonorrhea, Chlamydia, Syphilis, Tuberculosis, Campylobacter, Legionellosis, Salmonellosis, Shigellosis)*. For instance, Chlamydia and Syphilis, widespread sexually transmitted diseases in the TCDA, were ranked as 5th and 7th top communicable diseases for wastewater surveillance applications. Chlamydia and Syphilis are caused by bacteria *Chlamydia trachomatis* and *Treponema pallidum*, respectively. We successfully designed a bacterial wastewater surveillance workflow, optimized molecular microbiology laboratory methods to test and monitor *C. trachomatis* and *T. pallidum* in wastewater samples collected from TCDA, and developed a model that predicts per capita infection in the area (22). We used molecular tests to monitor polio (23) and we developed molecular and data analysis methods to track norovirus infections in TCDA (24).

In addition to CDWSRank, that focuses on ranking of reportable diseases, we developed high throughput sequencing and bioinformatics protocols to screen for emerging viral sequences that may be circulating the community (25, 26, 44). The protocols were applied to detect genomic sequences related to multiple human viruses excreted by the community (25, 26) that may be associated with reportable and non-reportable diseases. These results serve as a screening tool to guide further molecular analysis to confirm specific species identification in wastewater and clinical samples (Table 1).

**Table 1:** Examples of viral-related genomic sequences detected in Detroit Tri-County wastewater between 2020 and 2022 [more data and methods shown at Li et al. (25, 26)].

Viral family	Viral genus	Potentially associated illness
Adenoviridae	Mastadenovirus	Gastrointestinal, respiratory (common cold)
Astroviridae	Mamastrovirus	Gastrointestinal
Caliciviridae	Norovirus	Gastrointestinal
	Sapovirus	Gastrointestinal
Coronaviridae	Alphacoronavirus	Respiratory (common cold)
	Betacoronavirus	Respiratory (COVID-19)
Flaviviridae	Flavivirus	Vector-borne (Dengue, West-Nile)
	Hepacivirus	Bloodborne, direct contact (hepatitis C)
Hepeviridae	Orthohepevirus	Bloodborne (hepatitis E)
Hepadnaviridae	Orthohepadnavirus	Bloodborne, direct contact (hepatitis B)
Herpesviridae	Rhadinovirus	Direct contact (lymphoma, Kaposi sarcoma)
	Varicellovirus	Direct contact, respiratory (chicken pox, shingles)
	Simplexvirus	Direct contact (skin lesions)
	Roseolovirus	Direct contact (roseola rush)
	Lymphocryptovirus	Direct contact (mononucleosis)
	Cytomegalovirus	Direct contact (wide range of symptoms)
Matonaviridae	Rubivirus	Respiratory (rubella)
Orthomyxoviridae	Alphainfluenzavirus	Respiratory (influenza)
Papillomaviridae	Alphapapillomavirus	Direct contact (genital warts, cervical cancer)
	Nupapillomavirus	Direct contact (warts, skin lesions)
	Betapapillomavirus	Direct contact (warts, skin lesions)
	Gammapapillomavirus	Direct contact (warts, skin lesions)
	Mupapillomavirus	Direct contact (warts, skin lesions)
Parvoviridae	Bocaparvovirus	Respiratory
	Erythroparvovirus	Respiratory, direct contact (skin rush)
Picornaviridae	Hepatovirus	Gastrointestinal (hepatitis A)
	Parechovirus	Gastrointestinal, respiratory
	Enterovirus	Gastrointestinal, respiratory
	Cardiovirus	Zoonotic (myocarditis, encephalitis)
	Kobuvirus	Gastrointestinal
	Salivirus	Gastrointestinal
	Cosavirus	Gastrointestinal
Polyomaviridae	Betapolyomavirus	Respiratory (pathology in immunocompromised)
Poxviridae	Orthopoxvirus	Direct contact (skin lesions, lymphadenopathy)
	Parapoxvirus	Direct contact, zoonotic (skin lesions)
	Molluscipoxvirus	Direct contact (skin lesions)
Retroviridae	Lentivirus	Bloodborne, sexual (HIV-AIDS)
Togaviridae	Alphavirus	Vector-borne (Eastern equine encephalitis)

## 4 Discussion

### 4.1 Best practices

The partnership maintains strong communication with weekly meetings held among all stakeholders. These meetings typically follow an agenda that includes: Presentations of the latest wastewater laboratory measurements and other data as well as updates to the laboratory methods (MSU); Discussions of any challenges with sample collection or laboratory analysis (CDM Smith, GLWA, MSU); Announcements of any WRRF or collection system operational changes that might impact sample collection or results (Detroit Water and Sewerage District and GLWA); Discussions of the magnitude of the wastewater pathogen nucleic acid concentrations and their trends—by pathogen and location—and comparison with clinical datasets and social media trends (all stakeholders); Trends in clinical data and vaccination efforts (local health departments); How the wastewater data might be used to inform public health action (Detroit Health Department, Wayne County Health Department, Macomb County Health Department, and Oakland County Health Division); Thoughts on future directions for new wastewater targets (all stakeholders).

All of the data produced by this project are shared with the local health departments in TCDA and are used to make decisions regarding public health messaging for local awareness, targeted clinical testing, and increased vaccination efforts. The following publication shows one example, out of many (Figure 3), of local partners involvement in data analysis and application to allow early warning of COVID-19 in TCDA: *“Zhao L., Zou Y., Li Y., Miyani B., Spooner M., Gentry Z., Jacobi S., David R.E., Withington S., McFarlane S., Faust R., Sheets J., Kaye A., Broz J., Gosine A., Mobley P., Busch A.W.U., Norton J., Xagoraraki I. (2022) Five-week warning of COVID-19 peaks prior to the Omicron surge in Detroit, Michigan using wastewater surveillance, Science of the Total Environment, 844: 157040.”* Zhao, Li, Miyani, Spooner, Gentry and Jacobi are MSU students; Withington, McFarlane and Faust are epidemiologist in the local health department of TCDA (Detroit, Macomb and Oakland respectively); Sheets, Kaye and Brox, are with industry (CDM Smith); Gosine and Mobly are with the City of Detroit, and Bush and Norton are with industry (Great Lakes Water Authority).

Most of the data produced by this project are uploaded online weekly for general public access (27). Local, national and international media has covered the TCDA project and partnership.

### 4.2 Impact

Milestones of our academia-industry-government partnership in the TCDA have been described in 22 publications (co-authored by partners) which have been cited 777 times in Google Scholar as of December 14th, 2024. Among these publications, Miyani et al. (14) was awarded the 2022 Wesley W. Horner Award, a top national award from the American Society of Civil Engineers (ASCE) recognizing partnerships with industry. Our early warning methods for COVID-19 surges based on wastewater surveillance of SARS-CoV-2 (16), were referenced by the World Health Organization SARS-CoV-2 Variant Risk Evaluation Framework on August 30th, 2023 (28). These early warning methods were also modified and implemented in wastewater surveillance for respiratory syncytial virus (RSV) and Influenza A virus (IAV), where the researchers successfully identified early detections of RSV in Ontario, Canada (29), and researchers identified temporal trajectories of COVID-19, RSV and IAV in Northeastern China (30).

Our advanced statistical models based on wastewater measurements of SARS-CoV-2 to predict COVID-19 cases 5 weeks ahead of clinical reporting in the TCDA (17) were referenced by researchers that highlighted the potential non-linear effects that need to be accounted when establishing models to predict COVID-19 (31). Researchers also cited the five-week time lag in the TCDA between wastewater data and clinical data as well as factors potentially affecting time lags (31–36, 43).

Researchers cited the CDWSRank system by mentioning its significant contribution of developing a ranking system to address the knowledge gap of how to prioritize the future wastewater surveillance targets (37, 38). Researchers also specifically mentioned dengue virus and measles that can potentially monitored by wastewater surveillance (39), which were included in the ranking system proposed in Gentry et al. (21). Other researchers identified “practical feasibility” and “detectability of the pathogens” as primary factors to consider while selecting wastewater surveillance targets through referring to Gentry et al. (21) and Tiwari et al. (40). Researchers from the City of Houston Department of Health and Human Services referred to the ranking system, which we presented in Gentry et al. (21), as an outstanding example to expand wastewater surveillance program for infectious disease pathogens (41).

### 4.3 Looking forward

Our partnership has been actively engaging each stakeholder to identify newly emerging viral and bacterial targets in the TCDA and to develop wastewater surveillance methods for monitoring them. Looking forward, we are continuing wastewater surveillance in the TCDA for various viral diseases (such as SARS-CoV-2, influenza, RSV, and other respiratory, enteric and bloodborne viruses) as well as bacterial diseases including sexually transmitted and enteric illness. To establish baselines and test for changes in viral and bacterial pathogen diversity and mutations, we are conducting high throughput sequencing and bioinformatics-based screening of wastewater. Comparing genomic sequences helps us identify novel or potentially emerging infectious diseases to target with further testing. Additionally, we are continuing our effort to improve our prediction models by including multiple clinical and online datasets and analysis methods. For example, we plan to include social and behavioral databases, such social vulnerability data, EPA environmental justice screening and mapping, and google trends data; as well as mobility data.

Finally, we are planning to continue and expand our partnerships and include surveys to assess the needs of our extended stakeholders as well as addressing their potential concerns. For example, the issue of privacy is very important. We never collect wastewater from individual households, we look at the bigger scale and only collect regional wastewater. Investigating the population of a community, city or county at large provides valuable results to public health officials and no-one that has access to the results can pinpoint a specific household or a person (42).

Our work has demonstrated the value of a consortium comprised of academia, industry, and government, to collaborate and work toward shared goals. Our work has also demonstrated that environmental surveillance offers a pragmatic and reliable method for obtaining community composite samples (primarily municipal wastewater samples collected at a local treatment facility or a sewer manhole in a city neighborhood). For more targeted testing at critical locations, community composite samples may include aerosol, fomites, and wastewater samples collected on-site in hospitals, schools, residence halls, airports, event venues, or any public building. Our partnership will consider expanding our sampling methods and include alternative environmental samples. Combined with novel genomic testing and multidata process modeling, environmental surveillance provides accurate early warnings of disease outbreaks prior to detection at clinical settings.

## Data Availability

The original contributions presented in the study are included in the article/supplementary material, further inquiries can be directed to the corresponding author.
